# Miltefosine Suppresses Hepatic Steatosis by Activating AMPK Signal Pathway

**DOI:** 10.1371/journal.pone.0163667

**Published:** 2016-09-28

**Authors:** Ru Fang, Xudong Zhu, Yaqin Zhu, Xing Tong, Kexue Li, Hui Bai, Xiaoyu Li, Jingjing Ben, Hanwen Zhang, Qing Yang, Qi Chen

**Affiliations:** Atherosclerosis Research Center, Key Laboratory of Cardiovascular Disease and Molecular Intervention, Nanjing Medical University, Nanjing 210029, People’s Republic of China; East Tennessee State University, UNITED STATES

## Abstract

**Background and Purpose:**

It has been accepted that AMPK (Adenosine monophosphate–activated protein kinase) activation exhibits many beneficial effects on glucolipid metabolism. Lysophosphatidylcholine (LPC) is an important lysophospholipid which can improve blood glucose levels in diabetic mice and attenuate inflammation by activating AMPK signal pathway in macrophages. Synthetic alkylphospholipids (ALPs), such as miltefosine, is used as an alternate of LPC for the clinical application. Here, we investigated whether miltefosine could have an impact on hepatic steatosis and related metabolic disorders.

**Experimental Approach:**

Mice were fed with high fat diet (HFD) for 16 weeks to generate an obese model. Next, the obese mice were randomly divided into three groups: saline-treated and miltefosine-treated (2.5 or 5 mg/kg/d) groups. Miltefosine was intraperitoneally administrated into mice for additional 4 weeks plus HFD treatment.

**Key Results:**

It was shown that miltefosine treatment could substantially improve glucose metabolism, prevented hepatic lipid accumulation, and inhibited liver inflammation in HFD-fed mice by activating AMPK signal pathway. *In vitro*, miltefosine stimulated AMPKα phosphorylation both in time and dose dependent manner and decreased lipid accumulation in liver cells. When a specific AMPK inhibitor compound C was used to treat mice, the antagonistic effects of miltefosine on HFD-induced mouse hyperlipidaemia and liver steatosis were abolished. Treatment with miltefosine also dramatically inhibited the HFD-induced liver inflammation in mice.

**Conclusions and Implications:**

Here we demonstrated that miltefosine might be a new activator of AMPK signal pathway *in vivo* and *in vitro* and be useful for treatment of hepatic steatosis and related metabolic disorders.

## Introduction

Adenosine monophosphate–activated protein kinase (AMPK), a heterotrimeric enzyme consisting with catalytic α subunits and regulatory β and γ subunits, is a cellular energy sensor implicated in the regulation of lipid and glucose homeostasis [1–4]. Phosphorylation of Thr172 in α subunit leads to AMPK activation which is catalyzed by liver kinase B1 (LKB1), a tumor suppressor protein kinase, and calmodulin-dependent protein kinase kinase (CaMKK) [5–7]. Liver AMPK promotes fatty acid oxidation by phosphorylating and inactivating acetyl-CoA carboxylase (ACC) which leads to increased carnitinepalmitoyltransferase 1A (CPT1A) expression and fatty acid transport into the mitochondria for beta oxidation [8–10]. It also inhibits hepatic fatty acid synthesis by suppression of sterol regulatory element-binding protein 1C (SREBP1C) and fatty acid synthase (FAS) [11–13]. Thus, activation of AMPK appears to be an attractive therapeutic target for the treatment of hepatic steatosis and related metabolic disorders given the regulatory functions of AMPK on hepatic lipogenesis. Especially, novel AMPK activators are urgently needed [14–17].

Lysophosphatidylcholine (LPC) is an important lysophospholipid associated with many metabolic processes in the human body [18–20]. For example, LPC in plasma transports choline and fatty acids to tissues [21]. It can stimulate uptake of glucose by adipocytes by activation of glucose transporter type 4 (GLUT4) at the plasma membranes. Moreover, increased levels of LPC have been identified in the obese mouse serum and adipose tissue, which inhibits insulin resistance [22]. LPC also improves blood glucose levels in diabetic mice [23]. Besides, LPC is considered as of anti-inflammatory by activating AMPK signal pathway in macrophages [24]. However, application of LPC to the human being is limited by its very short half-life period and instability *in vivo*. Synthetic alkylphospholipids (ALPs) including edelfosine, miltefosine, and perifosine are used as the alternates of LPC for the clinical application. Among them, miltefosine is the prototype of the ALPs lacking the glycerol backbone and the alkyl chain being esterified directly to the phosphate group. It is an antitumor agent and has been used for effective treatment of leishmaniasis [25]. We hypothesized that miltefosine might have an impact on hepatic steatosis and related metabolic disorders given its properties mentioned above. The present study demonstrated that miltefosine could be a useful drug to antagonize high fat diet (HFD)-induced body weight increase, insulin resistance, and hepatic steatosis by activating AMPK signaling in mice.

## Materials and Methods

### Animals and reagents

C57BL/6 background mice were from the Jackson Laboratory. Animals were maintained under pathogen free conditions given free access to both food and water under temperature- and light-controlled animal facility with a 12-hours light/dark cycle, and the temperature was kept at 23±3°C with a relative humidity of 50%±10%. They were allowed to adapt to their food and environment for 1 week before starting the experiment. This study was carried out in strict accordance with the recommendations in the Guide for the Care and Use of Laboratory Animals of the National Institutes of Health, USA. The protocol was approved by the Research Animal Care Committee of Nanjing Medical University (permit number: NJMU/IUPAC 20140101–01). All surgery was performed under sodium pentobarbital anesthesia, and all efforts were made to minimize suffering. C57BL/6 male mice were fed by a HFD (60% kcal from fat, 20% kcal from CHO, 20% kcal from protein; Research Diets, New Brunswick, USA) or a nutrient-matched standard-fat chow diet (10% kcal from fat, 70% kcal from CHO, 20% kcal from protein; Research Diets) starting from age of 8 weeks. After 16 weeks, the obese mice were randomly divided into three groups: saline-treated and miltefosine-treated groups (2.5 mg/kg/d or 5 mg/kg/d). Miltefosine was purchased from Cayman Company (Ann Arbor,USA) and was intraperitoneally administrated into mice for additional 4 weeks plus HFD treatment. Physical condition of the animals was monitored every day through the experiment. No animal became severely ill or died at any time prior to the experiment endpoint. Mice were killed for the experiments and tissues were taken and frozen immediately in liquid nitrogen or fixed. For all the animal experiments, 7–10 mice were used for each group. Efforts were made to reduce the number of animals used and all procedures were as humane as possible.

We had a protocol in place for the early euthanasia/humane endpoints for animals who became severely ill/moribund during the experiment(s). Euthanasia as a matter of humane disposition occurs when continued existence is not an attractive option for the animal as perceived by the owner and veterinarian. When animals are plagued by disease that produces insurmountable suffering, it can be argued that continuing to live is worse for the animal than death or that the animal no longer has an interest in living. Clinical signs used to determine when to euthanize the animals include: quickly losing weight, loss of appetite for more than 3 days, too weak to get access to water and food, infection of organs which have no response to drug therapy, tumor which is bigger than 20 mm or causes infection and necrosis, tumor metastasis happen and so on. Mice were euthanized with carbon dioxide. Animals were euthanized by placing them into an empty chamber, and then the chamber was filled with carbon dioxide. Death was confirmed by examining the respiratory movement of thorax and faded eye color. Carbon dioxide exposure using a gradual fill method is less likely to cause pain due to nociceptor activation by carbonic acid prior to onset of unconsciousness. Carbon dioxide was continued for 1 more minute after complete cessation of respiration to ensure proper euthanasia.

### Glucose, body weight, serum insulin measurements and GTT

Blood glucose and body weight of mice were monitored weekly after fasting overnight. Glucose levels were measurements using the OneTouch Horizon Glucose Monitoring kit (LifeScan, Milpitas, USA) via tail vein blood sampling. The serum insulin levels were measured using an ELISA kit purchased from Millipore (Billerica,USA). Mice were intraperitoneally injected with 10% (w/v) glucose (1.0 g/kg) for glucose tolerance test (GTT) at the end of the experiment. After fasting for 12 hours (9 pm to 9 am), blood glucose was measured via tail vein blood sampling at 0, 30, 60 and 120 min, respectively. GTT were performed three times to prevent unnecessary stress that could interfere with results.

### Cell culture and treatments

Primary mouse peritoneal macrophages were cultured in DMEM (Hyclone, Logan, USA) containing 10% fetal bovine serum(FBS) (Gibco, Waltham, USA), 100 units/ml penicillin, 100 μg/ml streptomycin, and 5.5 mM D-glucose (normal glucose) as described previously [22]. After incubation for 24 hours and starvation overnight, cells were treated with LPS (lipopolysaccharide, Sigma-Aldrich St. Louis, MO, USA) and indicated concentrations of miltefosine for 12 hours. Hepatocytes were plated in 6 well culture plates with Williams medium E (Life Technologies, Inc., Grand Island, USA) containing 10% FBS, and incubated for 6 hours at 37°C in 5% CO_2_ as previously described [26]. After washing twice with phosphate buffered saline (PBS) and once with Williams medium E and incubated at 37°C for 24 hours, hepatocytes were used for the experiment. To identify the influence of high glucose on hepatic lipid accumulation, hepatocytes were incubated in serum-free Williams medium E containing either normal concentrations of glucose (5.5 mM D-glucose) or high concentrations of glucose (30 mM D-glucose) for 24 hours [27,28]. We employed a co-culture strategy using hepatocytes and macrophages to determine whether LPS induced pro-inflammatory cytokines from macrophages down-regulate AMPK signaling. Macrophages were isolated separately from 6 week old C57BL/6 mice and plated in transwell inserts containing 0.4 μm pore size membrane and incubated at 37°C. After incubation for 24 hours and starvation overnight, macrophages were treated with LPS and indicated concentrations of miltefosine for 18 hours. Then macrophages were washed by PBS for 3 times and plated in contact with the hepatocytes in Williams medium E and incubated at 37°C for 24 hours.

### Quantitative RT-PCR

Total RNA from the liver and cells were extracted using an RNAiso Plus (TaKaRa, Shiga, Japan). The quality of the RNA samples was examined at 260 and 280 nm with an UV spectrophotometer. Next, reverse transcriptase reactions were performed using commercial kits (Vazyme Biotech, Nanjing, China). Real-time reactions were performed using standard methods, and real-time PCR analysis was normalized to glyceraldehyde-3-phosphate dehydrogenase (GAPDH) (ABI Prism 7500 Sequence Detection System; Applied Biosystems, Foster city, USA).

### Immunoblot analysis

In brief, cells were washed three times with PBS and homogenized in protein lysis buffer containing 10% phosphatase inhibitors. Cell debris was removed by centrifugation at 12,000 х g for 15 minutes at 4°C. The supernatant (cell lysate) was used for immunoblot and measurements of lipid contents. Protein concentrations in cell lysates were measured using a Bio-Rad protein assay kit (Thermo Scientific, Rockford, USA). Proteins (20–70 μg) were separated by 10% SDS-PAGE, transferred to PVDF membrane and then blocked in blocking buffer (Tris-buffered saline, pH 7.6, 3% BSA and 0.05% Tween) for 1.5 hours. After incubation with primary antibodies diluted in blocking buffer at 4°C overnight and washing, detection was performed using appropriate secondary anti-IgG horseradish peroxidase conjugate (Santa Cruz Biotechnology, CA, USA) for 2 hours. Eventually, the membrane was washed and developed with Super-Signal chemiluminescent substrate (Thermo Scientific, Rockford, USA). The phosphorylation of LKB1, AMPK, ACC and SREBP1C were assessed by immunoblot with antibodies against phospho-Ser428 LKB1, phospho-Thr172 AMPK, phospho-Ser79 ACC and phospho-Ser372 SREBP1C (Cell Signaling Technology, Beverly, USA), as well as with antibodies against total LKB, AMPK, ACC (Cell Signaling Technology) and SREBP1C (NOVUS Biologicals, Littleton, USA) as loading controls. Antibody against FAS was purchased from Cell Signaling Technology. Antibodies against CPT1A and IRS2 were purchased from Millipore. Level of phosphorylation was normalized to the level of total protein and was expressed as relative phosphorylation to the basal or control level, which was normalized to that of GAPDH (Abcam, Cambridge, USA), and presented as the fold change relative to the control.

### Biochemical analyses

Concentrations of triglycerides and cholesterol in liver and serum were determined using the assay kits purchased from Nanjing Jiancheng Bioengineering Institute. Briefly, 20 μl of triglycerides and cholesterol standard or lipids extracted from tissues were added to a 96-well flat bottom polystyrene plate, and 200 μl of infinity triglycerides or cholesterol reagent (Nanjing Jiancheng Bioengineering Institute) was then added to the microplate. After incubation for 5 minutes, the optical density was read at 500 nm. Intracellular triglycerides and cholesterol levels were normalized to protein concentrations or tissue weight and expressed as mg lipid/g protein or mg lipid/g tissue [28,29]. Tissue and serum concentrations of CRP (C-reactive protein) were determined using a mouse CRP ELISA kit (Lanpai Biotechnology, Shanghai, China). Cellular activity of AMPK was analyzed using the AMPK Assay kit (Genmed Scientifics Inc., USA). In the presence of ATP, AMPK can phosphorylate the target substrate and generate phosphorylated polypeptides. Through the pyruvate kinase and lactate dehydrogenase reaction systems, reduced nicotinamide adenine dinucleotide turned to nicotinamide adenine dinucleotide. The reaction is terminated by the color change and measured spectrophotometrically at a wavelength of 340 nm. The concentration of AMPK in the samples is then determined by comparing the O.D. of the samples to the standard curve.

### Statistical analysis

All data are presented as the means ± S.E. Statistical analysis was performed by using GraphPad Prism 5.0 software (San Diego, USA). For comparisons among multiple groups, one-way ANOVA test was used. Comparison between two groups was performed by a two tailed unpaired Student’s test. All *in vivo* and *in vitro* experiments were performed blindly. A value of P < 0.05 was accepted as statistically significant.

## Results

### Miltefosine ameliorates HFD-deteriorated glucose metabolism in mice

**Fig 1:**
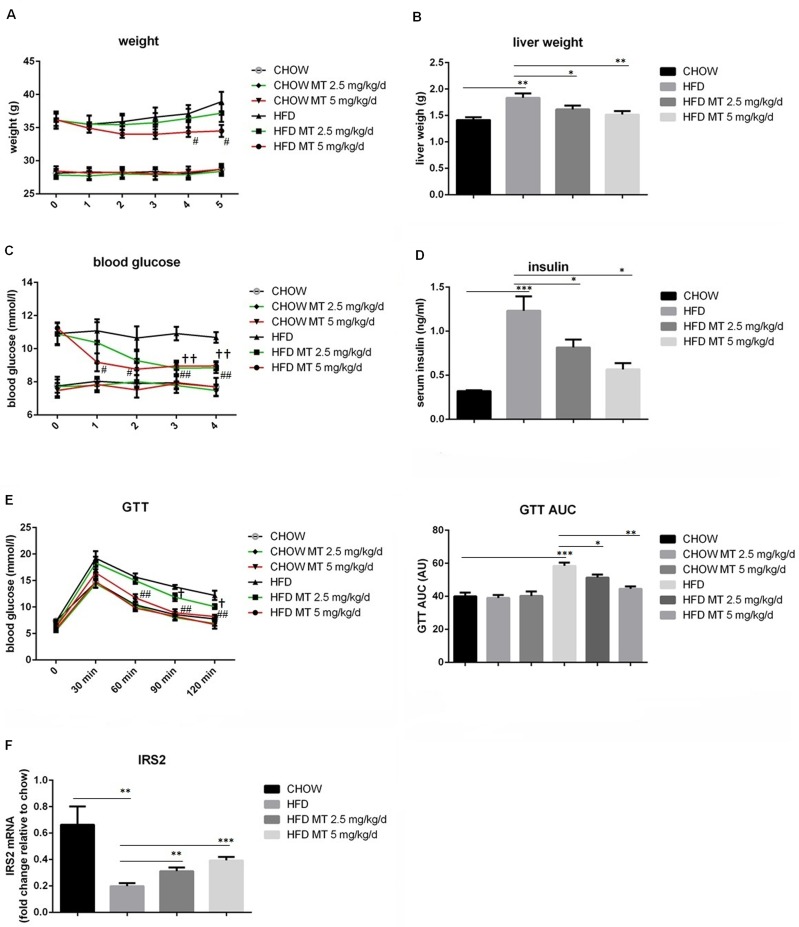
Effects of miltefosine on glucose metabolism in HFD mice. Mice were fed with a HFD or CD for 16 weeks. Miltefosine (2.5 or 5 mg/kg/d) was intraperitoneally administrated for additional 4 weeks plus HFD treatment (B-D). Body weights of the mice (A) were calculated every week. After sacrifice, liver tissues were removed to measure the weight (B). Blood levels of glucose (C) and insulin (D) were measured in fasted CD mice and HFD mice. (E-F) Glucose tolerance tests were performed by pretreatment with miltefosine or vehicle 2 hours before challenged with an administration of glucose (1g/kg) in mice (E). mRNA expression of IRS2 in the liver was measured by RT-PCR (F). n = 8. Data are expressed as the mean ± SEM. † P < 0.05, †† P < 0.01, HFD vs HFD MT 2.5 mg/kg/d; **#** P < 0.05, **##** P < 0.01, HFD vs HFD MT 5 mg/kg/d; * P < 0.05, **P < 0.01, ***P < 0.001.

In order to investigate the effect of miltefosine on glucolipid metabolism, mice were fed with a HFD for 16 weeks to generate an obese model and glucose metabolism and lipid metabolism were measured separately. Treatment with miltefosine had no obvious impact on body weight and blood glucose in CD-fed mice. However, it significantly ameliorated HFD-induced increases in body weight, liver weight, and blood glucose. Both doses of miltefosine (2.5 mg/kg/day and 5 mg/kg/day) exhibited therapeutic effect but a stronger effect was found in the high dose of group (Fig 1A–1C). HFD-induced high blood insulin was also dramatically attenuated by treatment with miltefosine (Fig 1D). Consistently, miltefosine was found to rescue impaired insulin response in HFD mice assayed by the GTT and measurement of expression level of insulin receptor S2 (IRS2) in the mouse liver (Fig 1E and 1F). These results suggest that administration of miltefosine improve HFD-deteriorated glucose metabolism in mice.

### Miltefosine inhibits HFD-induced hepatic lipid accumulation in mice

**Fig 2:**
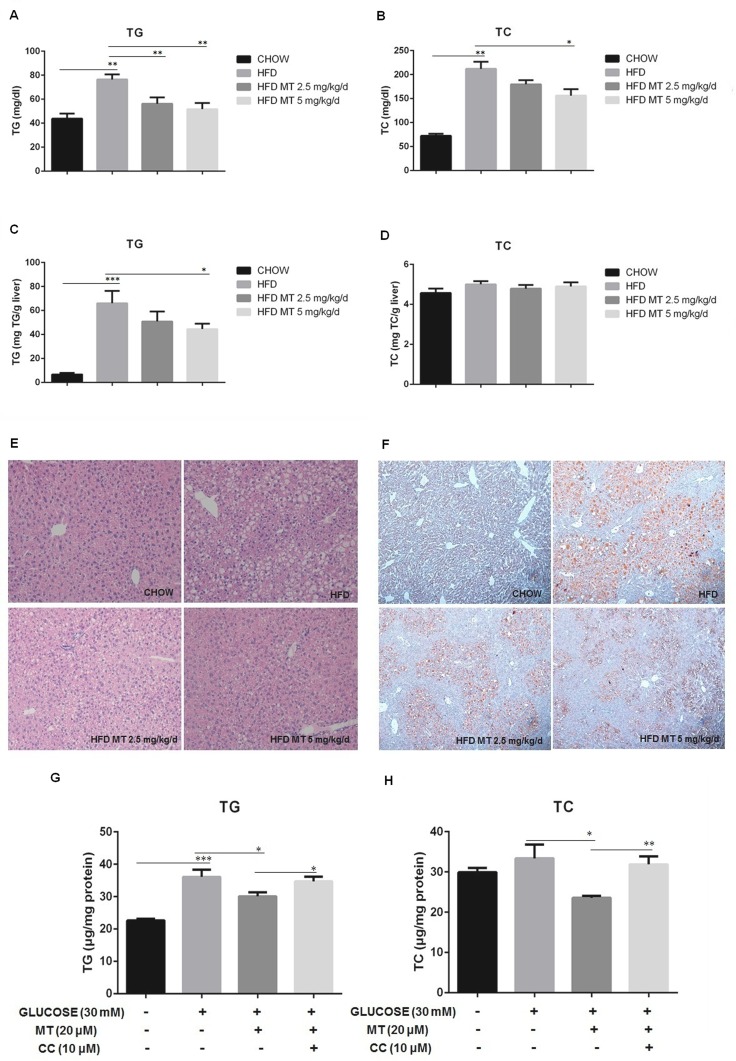
Effects of miltefosine administration on lipid accumulation and morphology of the liver in mice. (A-B) Serum triglycerides (A) and cholesterol levels (B) were measured in fasted mice. n = 8. (C-D) Liver triglycerides (C) and cholesterol (D) levels. n = 8. (E-F) Representative hematoxylin-eosin (HE) staining (E) and oil red staining (F) of the mouse liver. (G-H) Effects of compound C on intracellular triglycerides (G) and cholesterol (H) levels. n = 5. Data are expressed as the mean ± SEM. * P < 0.05, ** P < 0.01, *** P < 0.001.

We next examined effect of miltefosine on lipid metabolism in mice. As expected [30], HFD led to a dramatic hypertriglycemia and hypercholesterolemia in mice. Miltefosine treatment significantly reduced serum levels of triglycerides and cholesterol in HFD mice (Fig 2A and 2B). It is intriguing that HFD increased only level of hepatic triglycerides in mice. There was no obvious change in hepatic cholesterol level. Coordinately, miltefosine could reduce hepatic triglycerides level by 24.2% (2.5 mg/kg) to 33.3% (5 mg/kg) but had not obvious impact on hepatic cholesterol level in HFD mice (Fig 2C and 2D). Morphological analysis by H&E and oil red staining revealed that treatment with miltefosine eliminated excessive fat accumulation in hepatic intracellular vacuoles in HFD mice (Fig 2E and 2F).

To identify the influence of high glucose on hepatic lipid accumulation, we used high concentration of glucose (30 mM) to treat cultured primary mouse hepatocytes. This increased both levels of triglycerides and cholesterol in liver cells. Treatment with miltefosine significantly inhibited high glucose-induced increases in triglycerides and cholesterol in cultured liver cells (Fig 2G and 2H), suggesting an antagonizing effect of miltefosine on hepatic lipid accumulation induced by HFD.

### Miltefosine antagonizes HFD-impaired glucolipid metabolism by activating hepatic AMPK signal pathway in mice

**Fig 3:**
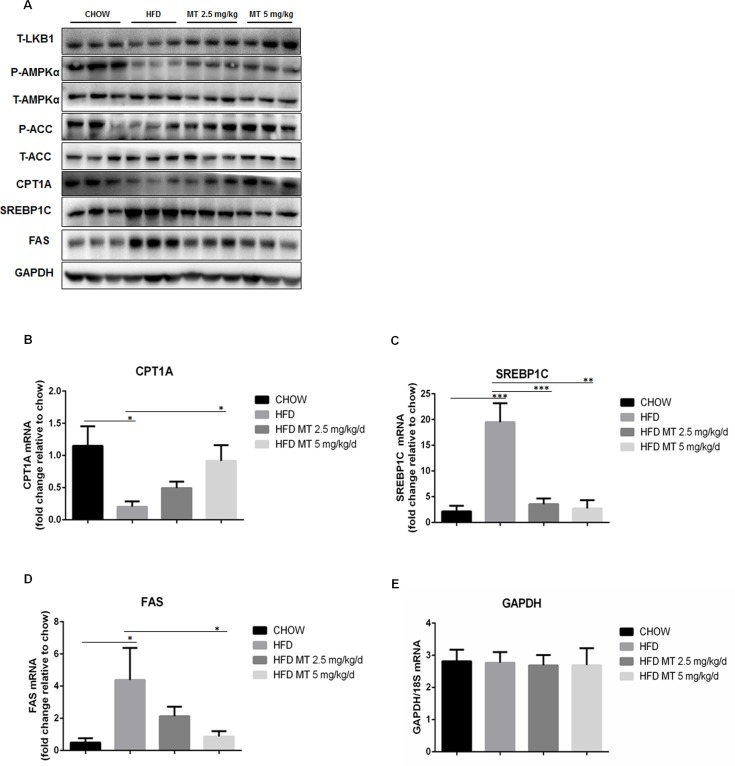
Effects of miltefosine on fatty acid metabolism-related signal molecules in mouse liver. (A) Western blot analysis of AMPK signal pathway in mouse liver lysates. n = 5. (B-E) Measurements of mRNA levels of AMPK signal pathway in the mouse liver by RT-PCR. n = 10. Data are expressed as the mean ± SEM. * P < 0.05, ** P < 0.01, *** P < 0.001.

To understand the mechanism of miltefosine inhibiting hepatic lipid accumulation, we examined the signal pathways associated with lipid metabolism in the liver. Western blot analysis revealed that HFD caused down-regulation of T-LKB1 and CPT1A and up-regulation of SREBP1C and FAS in the mouse liver. Phosphorylation of AMPKα and ACC were significantly suppressed in the HFD mouse liver. The HFD-induced changes in these signal molecules were substantially restored by miltefosine treatment (Fig 3A). These results were also corroborated by measurements of mRNA levels of CPT1A, SREBP1C, and FAS in the mouse liver (Fig 3B–3E), indicating that miltefosine may reverse HFD-induced suppression of fatty acid β-oxidation-associated signaling and activation of fatty acid synthesis-associated signaling by activating AMPK signaling in liver.

**Fig 4:**
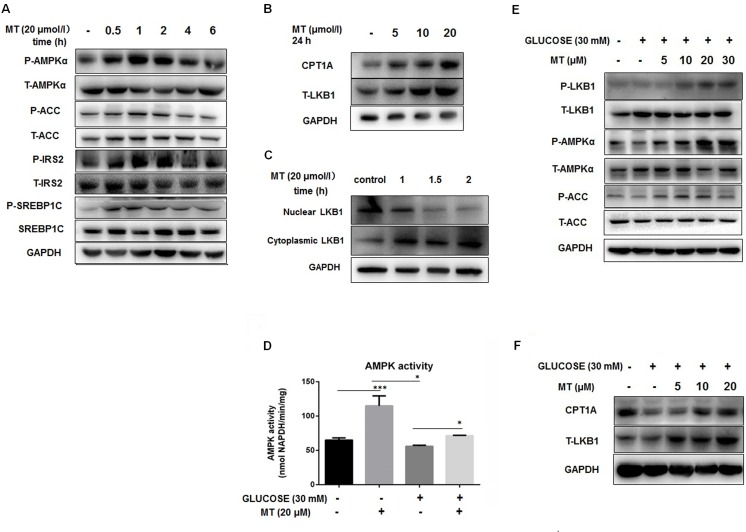
Effect of miltefosine on AMPK signal pathway in cultured primary mouse hepatocytes. Primary hepatocytes were cultured in serum-free medium overnight and treated with miltefosine for the indicated times. (A) Representative western blots of AMPKα, ACC, IRS2, and SREBP1C. (B) Effect of miltefosine on CPT1A and LKB1 expression. Cells were treated with miltefosine for 24 hours and cell lysates were quantified by western blot. (C) Nuclear and cytosolic levels of LKB1 were determined by western blot. (D) High glucose on AMPK activity in liver cells. (E) High glucose on AMPK signal pathway in liver cells. (F) High glucose on expression levels of CPT1A and LKB1 in liver cells. Data are expressed as the mean ± SEM. n = 5. * P < 0.05, *** P < 0.001.

We further measured AMPKα activity in cultured hepatocytes directly. We found that miltefosine stimulated strongly AMPKα phosphorylation in liver cells within 6 hours of incubation. It reached the maximum 1 hour after incubation. Similar effect of miltefosine was also found on the phosphorylation of ACC, IRS2, and SREBP1C (Fig 4A). Miltefosine increased expression of T-LKB1 and CPT1A (Fig 4B) and promoted an obvious cytosolic translocation of LKB1 in liver cells (Fig 4C). Hepatic AMPK was suppressed in the conditions of high glucose (Fig 4D). This could be reversed by addition of miltefosine into the cultured cells (Fig 4D and 4E). Again, in the presence of high glucose miltefosine could activate LKB1 and ACC and increased expressional levels of T-LKB1 and CPT1A in liver cells (Fig 4E and 4F).

**Fig 5:**
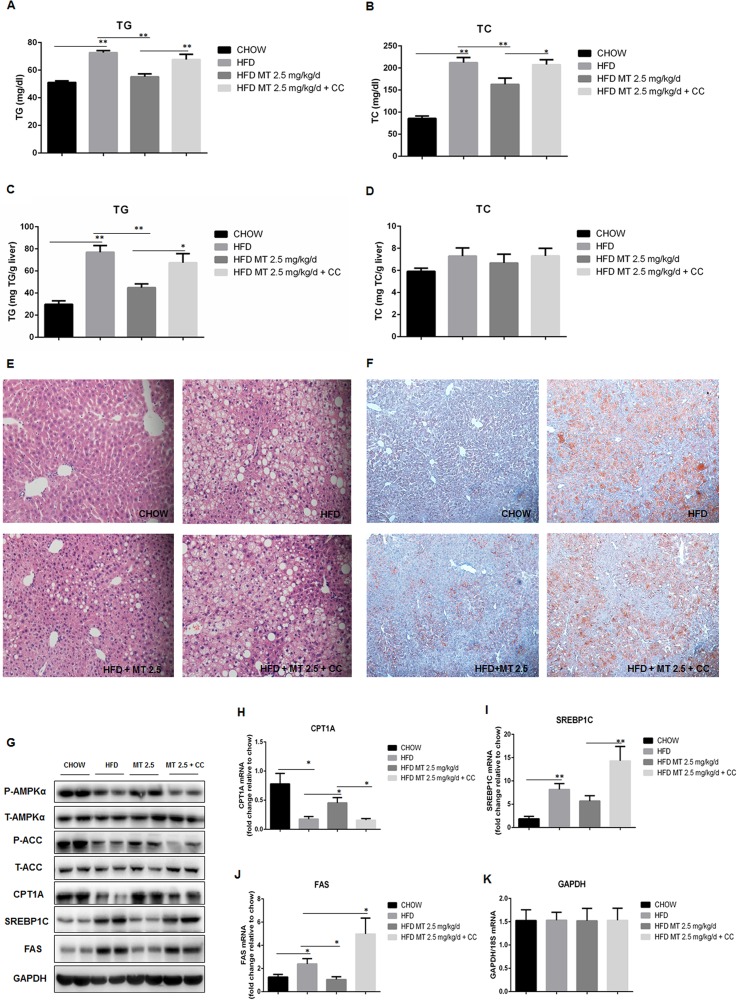
Effects of compound C on lipid metabolism and AMPK signal pathway in the mouse liver. (A-D) Levels of triglycerides (A, C) and cholesterol (B, D) in the mouse serum and liver were determined. n = 7. (E-F) Liver lipid accumulation was assessed by HE staining (E) and oil red staining (F). (G) Representative western blots of AMPK signal pathway in the mouse liver. n = 5. (H-J) RT-PCR measurements for mRNA levels of CPT1A (H), SREBP1C (I), FAS (J) and GAPDH (K) in the liver. n = 7. Data are expressed as the mean ± SEM. * P < 0.05, ** P < 0.01.

Compound C is a potent, reversible, and selective AMPK inhibitor exhibiting no significant inhibition of several structurally related kinases [31]. It was used to validate the stimulatory effect of miltefosine on AMPK signal pathway *in vivo* and *in vitro* in the present study. When mice were treated with 20 μg/g/d of compound C for 4 weeks, the antagonistic effects of miltefosine on HFD-induced mouse hyperlipidaemia and liver steatosis were abolished (Fig 5A–5F). Compound C could also completely prevent miltefosine’s effect by increasing triglycerides and cholesterol levels in cultured hepatocytes (Fig 2G and 2H). Consistently, compound C blocked all the activation of AMPK and ACC, up-regulation of CPT1A, and down-regulation of SREBP1C and FAS induced by miltefosine in the HFD-fed mouse liver (Fig 5G–5K), suggesting that compound C block the anti-liver steatosis effect of miltefosine via suppressing AMPK signal pathway.

### Miltefosine inhibits liver inflammation in mice

**Fig 6:**
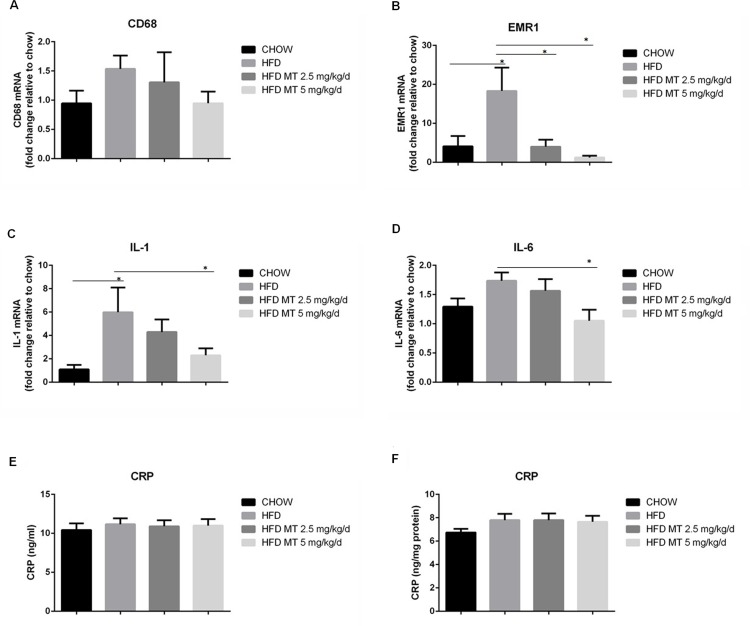
Miltefosine decreases macrophage infiltration and attenuates HFD-induced inflammation in the mouse liver tissue. (A-D) RT-PCR measurements for mRNA levels of CD68 (A), EMR1 (B), IL-1 (C), and IL-6 (D). n = 10. (E and F) Levels of CRP in mouse serum (E) and liver (F) were determined. n = 8. Data are expressed as the mean ± SEM. * P < 0.05.

Excessive lipid accumulation in liver is pro-inflammatory, which would impair liver functions. In the present study we found that increased levels of the macrophage markers, such as CD68 and EMR1, were strongly elicited in the mouse liver by HFD. Consistently, inflammatory cytokines including IL-1 and IL-6 were increased in HFD-fed mouse livers. Treatment with miltefosine dramatically inhibited the HFD-induced liver inflammation in mice (Fig 6A–6D). However, both HFD and miltefosine did not alter the levels of serum CRP and liver CRP in mice (Fig 6E and 6F).

**Fig 7:**
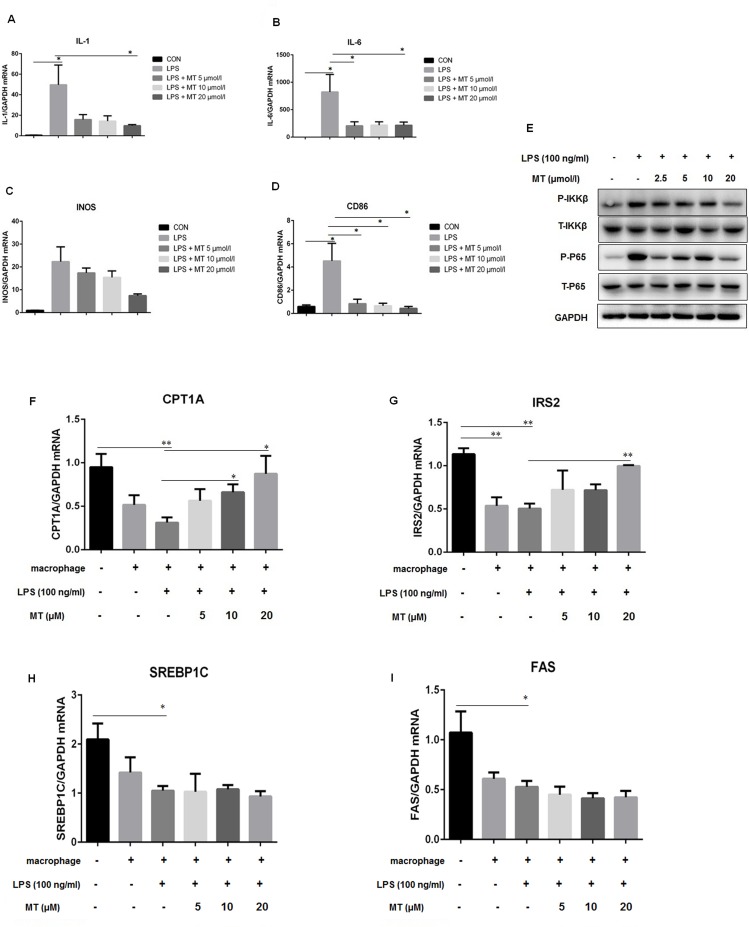
Effects of miltefosine on inflammation and NF-κb signaling in cultured cells. (A-D) Cultured mouse peritoneal macrophages were stimulated with LPS (100 ng/ml) and co-supplemented with indicated concentration of miltefosine. mRNA levels of IL-1 (A), IL-6 (B), INOS (C), and CD86 (D) in cells were measured by RT-PCR. n = 5. (E) Western blot of NF-κb signaling. n = 5. (F-I) Cultured primary hepatocytes were co-cultured with macrophages stimulated by LPS (100 ng/ml) and co-supplemented with the indicated concentration of miltefosine. mRNA levels of CPT1A (F), IRS2 (G), SREBP1C (H), and FAS (I) in cells were measured by RT-PCR after stimulation. n = 5. Data are expressed as the mean ± SEM. * P < 0.05, ** P < 0.01.

Mechanism underlying miltefosine inhibiting HFD-induced hepatic inflammation was further investigated by culturing mouse primary hepatocytes and peritoneal macrophages. We found that LPS caused a dramatic increase in pro-inflammatory cytokines IL-1 and IL-6 and M1 polarization signature molecules INOS and CD86 in macrophages. This was strongly inhibited by addition of miltefosine (Fig 7A–7D) which was via inhibition of NF-κb signaling in macrophages (Fig 7E). To examine influence of inflammation on liver lipid metabolism, liver cells were co-cultured with macrophages. As shown in Fig 7F–7I, addition of LPS into the co-culture system caused a down-regulation of CPT1A, IRS2, SREBP1C, and FAS in liver cells. Miltefosine strongly promoted an up-regulation of hepatic CPT1A and IRS2 but had no obvious impact on hepatic SREBP1C and FAS in the presence of macrophages plus LPS. These results suggest that miltefosine target inflammation-inhibited lipid oxidation.

**Fig 8:**
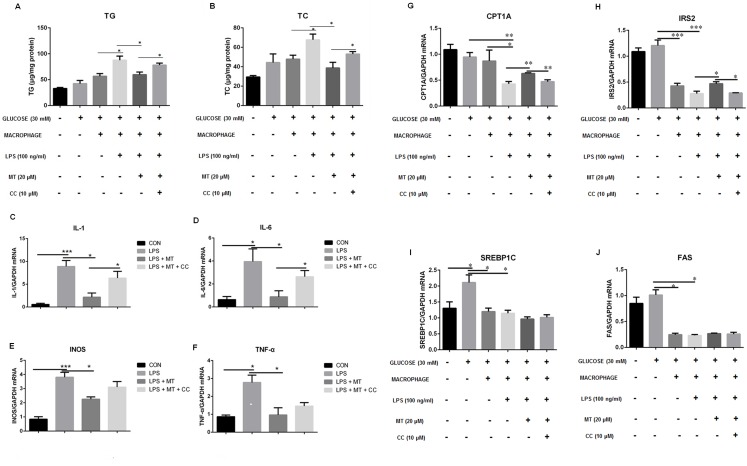
Effects of miltefosine on LPS-induced inflammation in co-cultured macrophages and hepatocytes. (A-B) Levels of triglycerides (A) and cholesterol (B) in cultured hepatocytes. (C-F) mRNA levels of IL-1 (C), IL-6 (D), INOS (E), and TNF-α (F) in macrophages were measured by RT-PCR. (G-J) mRNA levels of CPT1A (G), IRS2 (H), SREBP1C (I), and FAS (J) in hepatocytes were measured by RT-PCR. Data are expressed as the mean ± SEM. n = 5. * P < 0.05, ** P < 0.01, *** P < 0.001.

We also observed influence of high glucose on the anti-inflammatory effect of miltefosine. As shown in Fig 8A and 8B, presence of inflammatory macrophages resulted in a significant increase in lipids levels in cultured primary hepatocytes under high glucose conditions. This was dramatically inhibited by treatment with miltefosine. Compound C could block the lipid lowering effect of miltefosine. Under high glucose conditions, inflammatory macrophages also led to a down-regulation of CPT1A, IRS2, SREBP1C, and FAS in liver cells. Miltefosine exhibited an antagonistic effect only on CPT1A and IRS2 in this condition, which could be blocked by addition of compound C. No positive effect of miltefosine was found on SREBP1C and FAS (Fig 8G–8J). Therefore, these results suggest that anti-inflammation contribute to the mechanism of miltefosine antagonizing HFD-induced hepatic lipid accumulation.

## Discussion

Energy balance is maintained by many signaling pathways and "nutrient sensors" in the body. The AMPK acts an energy sensor to mediate adaptation to various cellular stressors from both the environment and nutrition and plays a key role in carbohydrate and fat metabolism to enable ATP conservation and synthesis [32]. Thus, the AMPK signaling pathway has been proposed as a useful target for treating type 2 diabetes mellitus (T2DM) and non-alcoholic fatty liver disease (NAFLD) [33]. In the present study we demonstrated that miltefosine may antagonize HFD-induced insulin resistance and liver steatosis by activating AMPK in mice.

Miltefosine is chemically a synthetic alkylphospholipid for the topical treatment of breast cancer metastasis and leishmaniasis and has been proven as a safe and well-tolerated drug. Up to now, no definite peptide target has been identified as the molecular pharmacological basis for its action. Miltefosine has been reported to selectively target proliferating (tumor) cells, inducing growth arrest and apoptosis [34]. In the present study, the dosage of miltefosine is one tenth of that for treatment of Leishmaniasis (2.5 mg/kg or 5 mg/kg) [35–37]. These doses of miltefosine did not caused obvious alteration in the proliferative activities of hepatocytes (data not showed). Furthermore, our *in vivo* and *in vitro* results demonstrated that the benefit effect of miltefosine on hepatic steatosis in HFD mice may be via activation of AMPK signal pathway.

Verma NK has reported that miltefosine inhibit insulin-induced IR-β, IRS-1, PI3K and Akt/PKB pathways in L6E9 skeletal muscle cells *in vitro* [38]. This is contradictory to our results that miltefosine could ameliorated HFD-induced insulin resistance and liver steatosis in mice. It is well known that miltefosine strongly inhibit PI3K/Akt/mTOR pathway in cells. Accumulative lines of evidence reveal that AMPK activation causes a down-regulation of PI3K/Akt/mTOR pathway in cancer cells [39,40], Thus, our data suggest that miltefosine may antagonize HFD-induced disorder in glycolipid metabolism and insulin resistance via activation of AMPK and inhibition of PI3K/AKt/mTOR pathway.

Although the detailed mechanism underlying miltefosine activating AMPK remains unknown, it is supposed that miltefosine may activate AMPK through lipid raft [41,42]. Lipid rafts serve as organizing centers for the assembly of signaling molecules, influencing membrane fluidity and membrane protein trafficking [43,44]. It has been reported that miltefosine is a modulator of lipid rafts which may be involved in the specificity and fidelity of AMPK signal transduction [45,46]. Interaction of miltefosine with lipid rafts may play a crucial role at the interface between membrane trafficking, AMPK activation, and reducing lipids. Therefore, it appears to be an interesting concept to utilize rafts as a new class of drug targets for AMPK activation.

As a novel AMPK activator, miltefosine was also shown of anti-inflammation. Similar anti-inflammatory effect has also been reported in perifosine, an ALP analogue [47]. It is intrigue that a significant inhibition on the infiltration of macrophages in HFD livers by miltefosine was only found in measurement of EMR1 but not in CD68 (Fig 6), Similar results were obtained by a CD68-staining immunochemical analysis (data not shown). This might be explained by that EMR1 is expressed in both kupffer cells and motile liver macrophages but CD68 is expressed only in kupffer cells [48]. However, measurements of both M1 cytokines (IL-1 and IL-6) and M1 signature molecules (INOS and CD86) suggest an anti-M1 polarization effect of miltefosine. Indeed, activation of AMPK serves as an anti-inflammatory target in modulating inflammatory responses [49–51]. We showed that anti-inflammatory effect of miltefosine is through inhibiting NF-κB signaling in macrophages. This might be attributed by activation of AMPK, because activation of AMPK signaling has been reported to inhibit NF-κB signaling [51–53]. Moreover, we found that compound C, a specific AMPK inhibitor, only partially blocked miltefosine’s inhibitory effect on inflammatory responses in macrophages (Fig 8C–8F), suggesting that other anti-inflammatory mechanisms beyond AMPK activation by miltefosine do not be ruled out. It is possible that modulating lipid raft property may endow miltefosine to a broader array of anti-inflammatory activities.

It seems to be advantageous for miltefosine to have different effects at different application concentrations. Its resisting catabolic degradation at the 40 mg/kg of dose results in itself accumulates in the cell and interfere with lipid-dependent survival signaling pathways, notably PI3K-Akt and Raf-Erk1/2, and de novo phospholipid biosynthesis [34]. Its activating AMPK signaling and inhibiting inflammation at less than 5 mg/kg/d could antagonize insulin resistance and liver steatosis induced by HFD in mice. This discovery certainly warrants further clinical trial but paves a path to use a market drug for treatment of hepatic steatosis and related metabolic disorders.

## Abbreviations

AMPK: Adenosine monophosphate–activated protein kinase
LKB1: liver kinase B1
CaMKKβ: calmodulin-dependent protein kinase kinase β
ACC: acetyl-CoA carboxylase
CPT1A: carnitinepalmitoyltransferase 1A
FAS: fatty acid synthase
SREBP1C: sterol regulatory element-binding protein 1C
T2DM: type 2 diabetes mellitus
LPC: Lysophosphatidylcholine
GLUT4: glucose transporter type 4
ALPs: alkylphospholipids
HFD: high fat diet
CD: chow diet
GTT: glucose tolerance test
GAPDH: glyceraldehyde-3-phosphate dehydrogenase
PMs: peritoneal macrophages
CRP: C-reactive protein
IRS2: insulin receptor S2
NAFLD: non-alcoholic fatty liver disease
